# Evaluation of the Effects of COVID-19 Infection and COVID-19 mRNA Vaccine on Ovarian Reserve

**DOI:** 10.3390/jcm15072614

**Published:** 2026-03-29

**Authors:** Zafer Basibuyuk, Ceren Cebi Basibuyuk, Seyma Okumus, Mahmut Oncul, Kutsiye Pelin Ocal

**Affiliations:** Department of Obstetrics and Gynecology, Istanbul University-Cerrahpaşa, Cerrahpaşa Medical Faculty, Istanbul 34098, Turkey; zaferbasibuyuk@gmail.com (Z.B.); okumusseymaa@gmail.com (S.O.); mahmutoncul@gmail.com (M.O.); drpelinocal@hotmail.com (K.P.O.)

**Keywords:** COVID-19, mRNA vaccine, ovarian reserve, AMH, menstrual cycle

## Abstract

**Objectives**: This study aimed to investigate whether COVID-19 infection or COVID-19 mRNA vaccination affects ovarian reserve and reproductive hormone profiles in reproductive-aged women. **Methods**: This retrospective longitudinal (before–after observational) single-center study included women aged 16–44 years who presented to a tertiary center between January 2021 and September 2023. Participants either had a confirmed COVID-19 infection by a positive polymerase chain reaction (PCR) test or had received two doses of a COVID-19 mRNA vaccine without prior infection. Women with available ovarian reserve parameters within six months of infection or vaccination were included. Anti-Müllerian hormone (AMH), follicle-stimulating hormone (FSH), luteinizing hormone (LH), estradiol (E_2_), prolactin (PRL), thyroid-stimulating hormone (TSH), total testosterone, and free testosterone levels were evaluated at baseline and reassessed six months later. Menstrual cycle characteristics were recorded. Parametric and non-parametric statistical tests were applied as appropriate. **Results**: No statistically significant differences were observed in AMH, FSH, LH, E2, PRL, TSH, total testosterone, or free testosterone levels before and after COVID-19 infection or vaccination (all *p* > 0.05). Comparisons between infection and vaccination groups across age subgroups (<25, 25–35, ≥35 years) revealed no significant differences in ovarian reserve parameters. Menstrual irregularities were reported in 38.0% of women following infection and 18.6% following vaccination. All reported menstrual changes were transient and resolved within six months. **Conclusions**: COVID-19 infection and mRNA vaccination were not associated with detrimental effects on ovarian reserve or reproductive hormone profiles. Although transient menstrual irregularities were observed, no long-term adverse reproductive impact was detected. Larger prospective studies are warranted to confirm these findings.

## 1. Introduction

COVID-19 is a systemic disease caused by SARS-CoV-2, a novel positive-sense single-stranded RNA coronavirus that primarily affects the respiratory system but also involves multiple organs [1]. Viral entry is mediated by the spike protein, which binds to angiotensin-converting enzyme 2 (ACE2) receptors and facilitates cellular infection. In addition to the respiratory epithelium, ACE2 is expressed in vascular endothelial cells, renal and intestinal tissues, and the central nervous system, providing a biological basis for the multisystem involvement observed in COVID-19 [2,3]. Notably, ACE2 expression within both the male and female reproductive systems has raised concerns regarding the potential impact of SARS-CoV-2 on gonadal function [4].

In women, the hypothalamic–pituitary–ovarian axis plays a central role in regulating the menstrual cycle and maintaining ovarian function [5]. Reports of increased menstrual disturbances among women recovering from COVID-19 have suggested several possible mechanisms, including endometrial hypoxia and inflammation, disruptions in hormonal regulation, and direct viral effects linked to ACE2 expression in ovarian tissue [4]. Although evidence remains limited, some studies have proposed that SARS-CoV-2 infection may adversely influence follicular development and granulosa cell function, both of which contribute to ovarian reserve. Data regarding the effect of COVID-19 on anti-Müllerian hormone (AMH)—a widely used biomarker of ovarian reserve—are inconsistent and inconclusive [6].

These biological considerations and clinical observations support the hypothesis that COVID-19 may exert potential effects on ovarian reserve. However, current evidence is insufficient, and findings are heterogeneous. Therefore, this study aimed to investigate the possible impact of COVID-19 infection on ovarian reserve in women.

## 2. Materials and Methods

### 2.1. Study Design and Setting

This retrospective longitudinal (before-after observational) single-center study included women aged 16–44 years who presented to a tertiary center between January 2021 and September 2023. No additional patients were enrolled after ethical approval; all data were obtained from previously recorded clinical records and were retrospectively extracted and analyzed following approval. Ovarian reserve parameters were evaluated 3–9 months (mean ~6 months) after PCR-confirmed COVID-19 infection or completion of a two-dose mRNA COVID-19 vaccination.

### 2.2. Participants

Women aged 16–44 years with either a PCR-positive COVID-19 infection or a documented two-dose mRNA vaccination and with available ovarian reserve tests within 6 months before infection or vaccination were screened.

### 2.3. Exclusion Criteria and Study Population

Participants were excluded if they had any of the following conditions: history of ovarian surgery, autoimmune disease, current or recent hormonal therapy (including oral contraceptive use), pregnancy, premature ovarian insufficiency, malignancy, hypothyroidism, use of immunosuppressive or cytotoxic therapy, assisted reproductive treatment, or missing baseline ovarian reserve data.

A total of 4270 patients with either confirmed COVID-19 infection or a history of COVID-19 mRNA vaccination were initially screened. Among these, patients with available baseline ovarian reserve parameters in the electronic medical records were identified (n = 261 for the infection group and n = 342 for the vaccination group). After applying the exclusion criteria, 122 patients in the infection group and 127 patients in the vaccination group were deemed eligible. During follow-up, 51 patients in the infection group and 30 patients in the vaccination group were excluded due to incomplete follow-up data. Ultimately, the study was completed with 71 patients in the infection group and 97 patients in the vaccination group.

### 2.4. Clinical and Menstrual Data

A structured interview captured age, gravidity, parity, BMI, medical history, smoking/alcohol habits, prior surgeries, medications, and PCOS diagnosis. Participants reported menstrual changes after infection or vaccination, classified as amenorrhea (≥3 months), oligomenorrhea (>35 days), hypomenorrhea (<2 pads/day), or menometrorrhagia (<21-day interval and >8 pads/day).

Menstrual characteristics were evaluated based on patient self-reports using standard clinical definitions (e.g., oligomenorrhea, hypomenorrhea, and menorrhagia). Baseline menstrual patterns prior to COVID-19 infection or vaccination were not systematically documented in the medical records.

### 2.5. Hormonal Measurements

Blood samples were obtained on cycle day 3; women with ≥3-month amenorrhea were evaluated regardless of cycle day. AMH, FSH, LH, estradiol, prolactin, and total and free testosterone were measured. COVID-19 PCR results and vaccination records were confirmed via electronic records. All baseline hormonal measurements were performed on cycle day 3. Laboratory analyses were conducted in the same center using a single standardized laboratory. The mean interval between baseline hormonal assessment and COVID-19 infection or vaccination was approximately 6 months (range: 3–9 months).

Potential sources of bias included reliance on electronic records for baseline data and self-reported menstrual changes, which may have introduced recall and information bias.

### 2.6. Statistical Analysis

Data were analyzed using SPSS v25. Descriptive statistics included means, standard deviations, medians, ranges, and frequencies. Statistical analyses were performed using appropriate parametric and non-parametric methods according to data distribution and variable type. Continuous variables were assessed for normality using visual (histograms) and analytical methods. As most hormonal parameters did not meet the assumptions of normal distribution, non-parametric tests were applied. Within-group comparisons of hormonal parameters before and after COVID-19 infection or vaccination were conducted using the Wilcoxon signed-rank test. Comparisons of hormonal changes across age groups were performed using the Kruskal–Wallis test. Associations between age and changes in hormonal parameters were evaluated using Spearman’s rank correlation analysis. Comparisons between two independent groups (infection vs. vaccination) for continuous variables were performed using the independent-sample *t*-test or the Mann–Whitney U test, as appropriate. Categorical variables, including menstrual irregularities, were compared using the Pearson chi-square test; Fisher’s exact test was considered when expected cell counts were less than five. In the statistical analysis, age, body mass index (BMI), PCOS status, and smoking status were considered as potential confounding variables and evaluated accordingly. All statistical tests were two-sided, and a *p*-value < 0.05 was considered statistically significant.

## 3. Results

**Figure 1:**
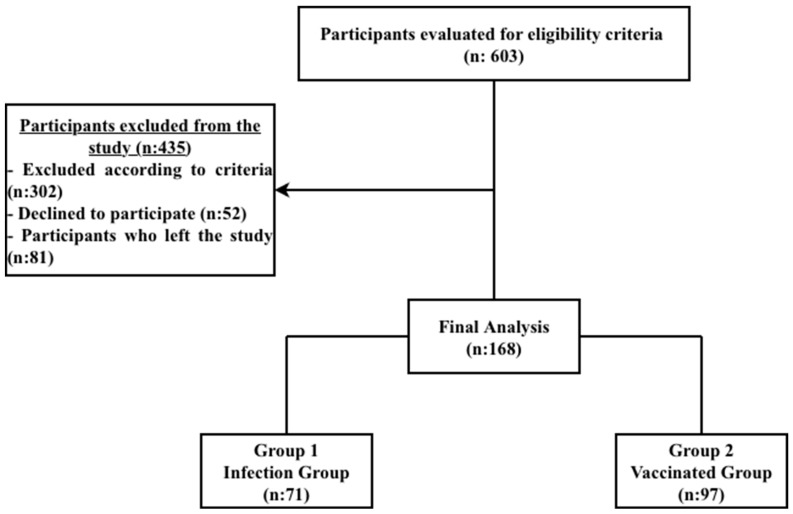
Flowchart of the study.

A total of 603 patients who applied to our center between the specified dates, had COVID-19 infection, or had received the COVID-19 mRNA vaccine were evaluated for eligibility (Figure 1). A total of 168 participants were included in the study. Patients were divided into 2 groups: Group 1 consisted of patients who had a COVID-19 infection (n:71), and Group 2 consisted of patients who had received the COVID-19 mRNA vaccine but did not have the infection (n:97).

Baseline demographic and clinical characteristics, including age, BMI, PCOS status, pregnancy status, parity, and smoking status, are presented in Table 1. The mean age was 30.5 ± 6.9 in Group 1 and 27.9 ± 7.6 in Group 2, with a significantly higher mean age in Group 1 (*p* = 0.026). This difference was considered normal, given that the elderly population is more susceptible to infection. No statistically significant differences were found between the two groups in terms of BMI, menstrual cycle length, duration of menstruation, amount of bleeding, smoking status, history of abdominal surgery, and presence of polycystic ovary syndrome.

Age differed statistically between the groups; however, the absolute difference was small and unlikely to be clinically significant.

Regarding obstetric history, the mean gravida in the infected group was 1.07 ± 1.32 (range 0–5), and the mean parity was 0.86 ± 1.02 (range 0–4). Patients in Group 1 had higher gravidity and parity than those in Group 2 (*p* = 0.00075 and 0.0017, respectively). This finding may be explained by the higher mean age of patients in Group 1.

Basal hormone parameters were measured in participants 6 months after vaccination or infection. Basal and 6-month hormone parameters for the infection and vaccination groups are shown in Table 2.

In both groups, there were no statistically significant changes in AMH, FSH, LH, estradiol (E2), prolactin (PRL), thyroid-stimulating hormone (TSH), total testosterone, or free testosterone levels over the six-month period (all *p* > 0.05).

A comparative assessment of hormonal changes showed that neither infection with COVID-19 nor vaccination was associated with significant changes in ovarian reserve markers or other reproductive and endocrine parameters during the study period. AMH levels remained stable in both groups, and no significant differences were observed before or after exposure.

Given the pivotal role of AMH in ovarian reserve assessment, no significant difference in AMH levels was observed in Group 1 before and after COVID-19 infection (3.70 ± 2.71 vs. 3.30 ± 2.71 ng/mL, *p* = 0.380). Similarly, no significant change in AMH concentrations was observed after vaccination in Group 2 (3.68 ± 2.60 vs. 3.47 ± 2.47 ng/mL, *p* = 0.540). These findings suggest that neither infection with COVID-19 nor vaccination has a measurable short-term impact on ovarian reserve, as assessed by AMH.

Participants were divided into three groups based on age: 16–25 years, 25–35 years, and 35–44 years. Values are presented as mean ± SD in Table 3. Changes in baseline ovarian parameters according to age were evaluated for both groups (infected and vaccinated) using the Kruskal–Wallis test. No statistically significant differences in hormonal changes were found between the two groups in all age groups. These findings indicate that neither COVID-19 infection nor vaccination causes age-related changes. Age was evaluated as a continuous variable, and all these hormonal change data were reanalyzed using Spearman correlation analysis. No statistically significant differences were found between the two groups in terms of age–vaccination/infection–hormonal parameter changes. This analysis is important to show that there was no bias in the age grouping of patients. When AMH changes were examined alone, no statistically significant differences were found across all age groups. Notably, this finding remained consistent even when women aged ≥35 to 44 years were included, supporting the conclusion that COVID-19 infection or vaccination does not have an age-related effect on AMH-related ovarian reserve dynamics.

Patients were asked about any menstrual irregularities they had experienced in the six months following infection or vaccination: amenorrhea: patients who did not menstruate for more than three months; oligomenorrhea: patients with a cycle length of more than 35 days; hypomenorrhea: patients with a bleeding amount of less than two pads per day; and menometrorrhagia: patients with a cycle length of less than 21 days and a bleeding amount of more than eight pads per day. The rates of infection and post-vaccination menstrual irregularities are shown in Table 4. The menstrual cycle patterns of the infection group (Group 1) and the vaccinated group (Group 2) were compared using the chi-square test (or Fisher’s exact test where appropriate).

Menstrual irregularities were observed in both study groups. However, menstrual irregularities were significantly more frequent in the infection group than in the vaccinated group (38.0% vs. 18.6%, *p* = 0.008). Amenorrhea was more prevalent among women with a history of COVID-19 infection than among vaccinated women (8.5% vs. 1.0%, *p* = 0.047). No statistically significant differences were found between the groups in terms of oligomenorrhea, hypomenorrhea, combined oligomenorrhea and hypomenorrhea, or menometrorrhagia (all *p* > 0.05). All reported menstrual disorders were temporary and resolved spontaneously within the six-month follow-up period.

## 4. Discussion

The present study demonstrates that neither COVID-19 infection nor COVID-19 mRNA vaccination results in significant changes in serum AMH levels or other reproductive hormone parameters, and neither exposure appears to adversely affect ovarian reserve. These findings are consistent with a growing body of literature indicating that COVID-19 infection and vaccination do not have a detrimental effect on ovarian function.

Madendağ et al., in a prospective study including 132 women, reported no statistically significant differences in AMH, FSH, LH, or estradiol concentrations when comparing measurements obtained before and after COVID-19 infection [7]. Similarly, in a retrospective cross-sectional study conducted by Kezhen Li et al., which included 177 women (with hormonal data available for 91 participants), AMH concentrations before and after COVID-19 infection were comparable to those observed in the control group [8].

Regarding vaccination-related studies, Ata et al. evaluated the effect of COVID-19 mRNA vaccination on ovarian reserve and found no significant changes in AMH or antral follicle count following vaccination [9]. Likewise, Mohr-Sasson et al., in a prospective study involving 129 women, reported comparable AMH levels measured before vaccination and three months after the second vaccine dose [10]. Orvieto et al. further demonstrated that ovarian reserve parameters and ovarian response during in vitro fertilization cycles were not adversely affected following COVID-19 vaccination [11]. Collectively, these findings are in agreement with our results, which showed no significant changes in AMH or other ovarian reserve and reproductive hormone parameters at the six-month follow-up after either COVID-19 infection or vaccination.

In contrast to these reports, Ding et al. conducted an observational study including 78 women with COVID-19 infection and 151 control participants and reported significantly lower AMH levels and higher FSH levels in the infected group [12]. However, it should be noted that this study primarily included hospitalized patients with severe COVID-19, suggesting that systemic inflammation, stress, and severe illness may have contributed to the observed hormonal alterations rather than a direct effect of COVID-19 on ovarian reserve.

Importantly, the consistency of our findings across age groups and when age was analyzed as a continuous variable using Spearman correlation analysis indicates that age did not significantly modify the hormonal response to either COVID-19 infection or COVID-19 vaccination in our cohort. Although age is a major determinant of baseline ovarian reserve, we found no evidence of an age-dependent adverse effect in the context of COVID-19 exposure or immunization.

In addition to hormonal outcomes, transient menstrual irregularities were observed in both study groups. Menstrual disturbances were significantly more frequent in the infection group than in the vaccinated group (38.0% vs. 18.6%, *p* = 0.008). Amenorrhea was also observed more commonly among women with prior COVID-19 infection compared with vaccinated women (8.5% vs. 1.0%, *p* = 0.047). No statistically significant differences were detected between groups regarding oligomenorrhea, hypomenorrhea, combined oligomenorrhea and hypomenorrhea, or menometrorrhagia. All reported menstrual disturbances were transient and resolved spontaneously within the six-month follow-up period.

Several mechanisms have been proposed to explain these transient menstrual changes. One hypothesis involves stress-related disruption of the hypothalamic–pituitary–ovarian axis. Another theory suggests that the immune response triggered by COVID-19 infection may influence immune cell activity within the endometrium, leading to temporary alterations in menstrual bleeding patterns or early endometrial shedding. Additionally, COVID-19 may affect the renin–angiotensin system through downregulation of ACE2 receptors, potentially resulting in increased angiotensin-II activity, spiral artery vasoconstriction, and subsequent menstrual irregularities.

Our findings are consistent with previous studies evaluating menstrual changes following COVID-19 infection. Li et al. reported menstrual volume changes, predominantly decreases, in 25% of women and cycle length changes, predominantly prolongation, in 28% of patients following COVID-19 infection, with 84–99% of women returning to their normal menstrual patterns within one to two months after discharge [8]. Similar observations were reported by Madendağ et al. and Ding et al., who noted that menstrual volume reductions normalized within three months after recovery and that these changes generally resolved within a few menstrual cycles [7,12].

Regarding the COVID-19 vaccine, a large meta-analysis by Nazir et al., involving 78,138 participants from 14 studies, reported a 52% incidence of menstrual irregularities after vaccination. However, this meta-analysis also concluded that these menstrual irregularities were temporary [13]. Consistent with these findings, studies by Ata et al. and Nazzal et al. suggested that both COVID-19 infection and vaccination may be associated with transient menstrual changes, with infection-related effects generally being more pronounced and longer-lasting, whereas vaccine-related changes were minimal and resolved within a few cycles [9,14].

Overall, our findings support the growing evidence that no short-term association was observed between COVID-19 infection or vaccination and ovarian reserve parameters. However, given the retrospective design and limited sample size, these findings should be interpreted with caution. Although transient menstrual disturbances may occur, these changes appear to be self-limiting and resolve spontaneously within a few months.

The most important limitation of this study is the absence of antral follicle count (AFC) data, a key ultrasonographic marker of ovarian reserve. Due to pandemic-related restrictions and limited access to elective clinical evaluations, AFC measurements could not be obtained. As a result, ovarian reserve assessment relied solely on hormonal markers (AMH, FSH, and LH), which, although widely used, may not fully capture the functional and dynamic aspects of ovarian physiology. This limitation may have reduced the ability to detect subtle changes in ovarian function.

In addition, COVID-19 infection severity could not be stratified due to limitations in the available clinical records, which may influence the interpretation of the findings. The relatively small sample size also represents a limitation, as post hoc analysis based on the observed effect size for ΔAMH (Cohen’s d = 0.23) suggested that approximately 295 participants per group would be required to achieve 80% power. Therefore, the study may have been underpowered to detect small effect sizes, and the absence of statistically significant differences should be interpreted with caution. Nevertheless, the small observed effect size suggests that any potential impact is likely limited in magnitude and unlikely to be clinically meaningful.

Furthermore, baseline menstrual characteristics were not systematically recorded, and menstrual changes were assessed based on patient-reported data; therefore, these findings should be considered exploratory. Other potential confounding factors, including psychological stress, lifestyle or weight changes, and time since vaccination, could not be fully evaluated due to the retrospective design, and residual confounding cannot be excluded.

Despite these limitations, the study is strengthened by its single-center design, the use of a standardized laboratory, and the consistency of the findings with the existing literature.

## 5. Conclusions

In conclusion, no short-term association was observed between COVID-19 infection or vaccination and ovarian reserve parameters. However, given the retrospective design and limited sample size, these findings should be interpreted with caution. Although transient menstrual irregularities were observed in both groups—more frequently following COVID-19 infection than vaccination—these changes were self-limited and resolved spontaneously during follow-up. Our results are consistent with the existing literature and provide further reassurance regarding the short-term reproductive endocrine safety of both COVID-19 infection and vaccination. These data support counseling women of reproductive age for whom COVID-19 infection and mRNA vaccination do not appear to have a detrimental impact on ovarian reserve or long-term menstrual function, while emphasizing the transient and reversible nature of any observed menstrual disturbances.

## Figures and Tables

**Table 1:** Demographic characteristics of the infection and vaccination groups.

	Group 1-Infection Group (n:71)		Group 2-Vaccinated Group (n:97)		
Variables	Mean ± SD	Median (Min–Max)	Mean ± SD	Median (Min–Max)	*p*
Age(years)	30.5 ± 6.9	31 (16–44)	27.9 ± 7.6	25 (16–44)	**0.026** ^a^
BMI	24.6 ± 4.2	25.2 (16–37)	23.5 ± 3.2	22.9 (17.7–34.1)	0.068 ^a^
Gravidity	1.07 ± 1.3	0 (0–5)	0.45 ± 0.8	0 (0–4)	**0.00075** ^a^
Parity	0.86 ± 1.0	0 (0–5)	0.40 ± 0.8	0 (0–1)	**0.0017** ^a^
Menstrual cycle length (days)	34.8 ± 14.5	28 (26–90)	33.8 ± 10.7	30 (25–90)	0.604 ^a^
Menstrual duration (days)	5.3 ± 1.7	5 (1–10)	5.4 ± 1.7	5 (2–10)	0.624 ^a^
Amount of bleeding (pads/day)	3.7 ± 1.4	4 (1–8)	3.9 ± 1.3	4 (1–10)	0.286 ^a^
	**n**	**%**	**n**	**%**	
Polycystic ovary syndrome	11	15.5	15	15.4	1.0 ^b^
Previous abdominal surgery	18	25.4	14	14.4	0.114 ^b^
Smoking status					0.537 ^b^
Present	18	25.4	30	30.9	
Absent	53	74.6	67	69.1	

a: independent-sample *t*-test. b: chi-square test.

**Table 2:** Comparison of hormonal parameters in the infection and vaccination groups.

Variables	Group	Mean ± SD		*p* ^c^
		Before	After	
**AMH (ng/mL)**	**1 (n = 71)**	3.70 ± 2.71	3.30 ± 2.71	0.380
	2 (n = 97)	3.68 ± 2.60	3.47 ± 2.47	0.540
**FSH (mIU/mL)**	1 (n = 71)	6.44 ± 2.53	6.60 ± 2.65	0.721
	2 (n = 97)	6.11 ± 2.21	6.36 ± 2.44	0.454
**LH (mIU/mL)**	1 (n = 71)	8.25 ± 4.20	8.33 ± 4.40	0.918
	2 (n = 97)	7.03 ± 3.54	7.07 ± 3.26	0.951
**E2 (pg/mL)**	1 (n = 71)	54.05 ± 32.92	52.58 ± 25.27	0.773
	2 (n = 97)	52.22 ± 37.82	49.54 ± 27.69	0.579
**PRL (ng/mL)**	1 (n = 71)	20.50 ± 11.16	20.80 ± 8.70	0.858
	2 (n = 97)	22.12 ± 11.07	21.98 ± 8.82	0.925
**TSH (µIU/mL)**	1 (n = 71)	2.27 ± 1.44	2.07 ± 1.18	0.357
	2 (n = 97)	2.19 ± 0.98	2.09 ± 0.91	0.471
**Total Testosterone (ng/dL)**	1 (n = 71)	42.76 ± 17.01	41.05 ± 15.06	0.691
	2 (n = 97)	38.06 ± 12.55	36.97 ± 13.56	0.681
**Free Testosterone (pg/mL)**	1 (n = 71)	2.30 ± 1.23	2.08 ± 0.92	0.712
	2 (n = 97)	1.80 ± 0.66	1.81 ± 0.72	0.977

c: Wilcoxon signed-rank test.

**Table 3:** Comparison of changes in hormone parameters in the two groups according to age.

Variables	Group		Mean ± SD		*p* ^d^
		≤25 Age	25–35 Age	≥35–44 Age	
**Delta AMH (ng/mL)**	1 (n:71)	0.4 ± 0.9	0.4 ± 0.9	0.4 ± 0.4	>0.05
	2 (n:97)	0.2 ± 0.7	0.1 ± 0.7	0.3 ± 0.6	>0.05
**Delta FSH (mIU/mL)**	1 (n:71)	0.6 ± 1.4	0.04 ± 3.4	−0.2 ± 3.2	>0.05
	2 (n:97)	0.1 ± 0.4	−0.06 ± 1.2	0.8 ± 3.0	>0.05
**Delta LH (mIU/mL)**	1 (n:71)	−0.8 ± 2.3	0.4 ± 3.1	0.6 ± 3.0	>0.05
	2 (n:97)	−0.1 ± 3.3	0.04 ± 2.6	0.3 ± 2.3	>0.05
**Delta E2 (pg/mL)**	1 (n:71)	2.9 ± 19.0	−1.6 ± 18.3	6.9 ± 58.0	>0.05
	2 (n:97)	0.5 ± 44.9	1.4 ± 28.8	9.4 ± 46.3	>0.05
**Delta PRL (ng/mL)**	1 (n:71)	−0.2 ± 13.3	0.1 ± 19.1	1.6 ± 8.7	>0.05
	2 (n:97)	−2.1 ± 11.6	1.6 ± 12.3	3.6 ± 8.7	>0.05
**Delta TSH (µIU/mL)**	1 (n:71)	0.6 ± 1.7	−0.04 ± 1.0	0.2 ± 1.4	>0.05
	2 (n:97)	0.1 ± 1.1	0.1 ± 1.0	0.04 ± 1.0	>0.05

d: Kruskal–Wallis test.

**Table 4:** Rates of menstrual irregularities following infection and vaccination.

Menstrual Pattern	Group 1 (n = 71)		Group 2 (n = 97)		*p* ^b^
	n	%	n	%	
**Any menstrual irregularity**	27	38	18	18.5	***0.008***
**Amenorrhea**	6	8.4	1	1	***0.047***
**Oligomenorrhea**	5	7	5	5.1	0.857
**Hypomenorrhea**	6	8.4	2	2.1	0.120
**Oligomenorrhea + hypomenorrhea**	6	8.4	7	7.2	0.997
**Menometrorrhagia**	4	5.6	3	3.1	0.672

b: Chi-square test (categorical variables were compared using the chi-square test or Fisher’s exact test, as appropriate).

## Data Availability

The data that support the findings of this study are available upon reasonable request from the corresponding author.

## Abbreviations

mRNA	Messenger RNA
AMH	Anti-Müllerian hormone
E2	Estradiol
FSH	Follicle-stimulating hormone
LH	Luteinizing hormone
PCR	Polymerase chain reaction
PRL	Prolactin
TSH	Thyroid-stimulating hormone
ACE2	Angiotensin-converting enzyme 2
BMI	Body mass index
PCOS	Polycystic ovary syndrome
